# Different Varieties of Water Caltrop (*Trapa bispinosa*) Starch: Physicochemical Properties and Digestibility Modulated by Its Multi-Scale Structure

**DOI:** 10.3390/foods14244304

**Published:** 2025-12-14

**Authors:** Tengfei Ma, Qiong Wu, Yuyang Yuan, Xiaoxin Chen, Qinlu Lin, Huaxi Xiao, Jiangtao Li, Wenfang Han

**Affiliations:** 1 National Engineering Research Center of Rice and Byproduct Deep Processing, College of Food Science and Engineering, Central South University of Forestry and Technology, Changsha 410004, China; fh2974018652@163.com (T.M.); 18907470141@163.com (Y.Y.); 13450121026@163.com (X.C.); linql0403@126.com (Q.L.); xiaoxijingjing@163.com (H.X.); 2 Shantou Technician College, Shantou 515000, China; wq662778@163.com

**Keywords:** water caltrop, starch, multi-scale structure, physicochemical properties, digestibility

## Abstract

This study assessed the physicochemical properties and digestibility of starches derived from five varieties of water caltrop, focusing on their multi-scale structure. Water caltrop starch granules exhibited round, oval, or polygonal shapes with smooth surfaces, exhibiting unimodal particle size distributions and A-, C-, or C/A-type crystal patterns. *T.qR‘Green’* exhibited the highest amylose content (30.93%), the lowest peak viscosity and breakdown, and the highest setback. *T.bR‘Green’* had the highest crystallinity (29.04%) and endothermic enthalpy (15.39 J/g), with a more ordered internal structure. *T.bR‘Red’* had the lowest crystallinity (24.94%), gelatinization temperature, and endothermic enthalpy (8.08 J/g), while showing the highest peak viscosity and breakdown, the lowest setback, and the highest resistant starch content (47.2%), thus possessing stronger resistance to digestion. Pearson correlation analysis revealed that the thermal properties of water caltrop starches were mainly influenced by the amylopectin B-chains and short-range order, while pasting properties were mainly affected by amylopectin B-chains and crystallinity. Amylose content positively influenced solubility but negatively affected swelling power. Additionally, water caltrop starch digestibility showed a negative correlation with granule size and short-range order. These findings indicated the significant impact of starch multi-scale structure on physicochemical properties and digestibility.

## 1. Introduction

Water caltrop (*Trapa bispinosa*) is an annual aquatic plant with floating leaves, classified within the Lythraceae family and Trapa genus. It is commonly found in freshwater wetlands, lakes, ponds, and stagnant river segments across tropical to temperate zones [1]. Its fruit, rich in carbohydrates (60–70% on a dry basis), proteins, vitamins, and various bioactive components [2,3], provides both nutritional and medicinal value. Starch, the predominant energy reserve compound in water caltrop, comprises more than 60% of its dry mass and possesses a complex hierarchical organization involving granular morphology, crystalline structure, and chain length distribution. These structural features play a crucial role in governing various physicochemical characteristics of starch, such as gelatinization, retrogradation, swelling power, and digestibility [4]. These properties further influence its application potential in the food industry (e.g., as fat substitutes and edible films) and non-food sectors (e.g., biodegradable materials and drug delivery systems) [5,6,7]. In addition, compared to other starch sources such as potatoes and rice, the cultivation process of water caltrops can reduce land resource occupation. Moreover, it is widely available and economical, making it a suitable candidate for supplying a large amount of starch.

While the basic characteristics of water caltrop starch have been partially understood in recent years, existing research has predominantly examined individual varieties [2,8,9,10]. As a result, the influence of varietal distinctions on starch composition and performance has not been comprehensively investigated. It is worth noting that considerable diversity in starch properties within different varieties of the same plant species has been extensively reported. Yao et al. [11] found that there were significant differences in particle size, crystallinity, and digestion characteristics among different varieties of mung bean starch. Similarly, Zhang et al. [12] illustrated cultivar-specific variances in starch granule size, crystalline structure, and pasting properties of litchi seed starch. Kovač et al. [13] documented marked distinctions in amylose levels, solubility, and swelling power among starch samples sourced from diverse potato cultivars. These findings suggest that genetic background influences starch biosynthesis pathways, leading to differences in granule morphology, molecular order, and functional properties. Nevertheless, the impact of varietal specificity on the intricate interplay between the structure and function of starch in water caltrop, a starch-rich crop, has not been investigated.

This research centered on five water caltrop varieties, specifically examining the extraction of starch from their flesh. Multiple characterization techniques, including scanning electron microscopy (SEM), laser particle size analysis, small-angle X-ray scattering (SAXS), X-ray diffraction (XRD), Fourier transform infrared spectroscopy (FTIR), and solid-state nuclear magnetic resonance (NMR), were employed to analyze the multi-scale structure of water caltrop starch. Differential scanning calorimetry (DSC) and in vitro digestion models were further used to examine gelatinization and digestive properties. By highlighting the varietal differences in the structure, properties, and functions of starch, this work establishes a theoretical foundation for the tailored utilization of water caltrop starch, such as in customized processing and the development of high-value products. Moreover, it contributes to a multidimensional evaluation system for aquatic plant starch resources.

## 2. Materials and Methods

### 2.1. Materials

Fruits of *T.bR‘Green’* were harvested from Nantong City, China, while those of *T.bR‘Red’* were obtained from Huaian City, China. Fruits of *T.a* and *T.qR‘Red’* were procured from Jiaxing City, China, and those of *T.qR‘Green’* were collected in Suqian City, China. Glucose oxidase/peroxidase (GOPOD) for determining glucose content was sourced from Megazyme International Ireland (Bray Business Park, Bray, Co., Wicklow, Ireland). All other reagents used were of analytical grade.

### 2.2. Isolation of Water Caltrop Starch

Peel the water caltrop fruit to leave the flesh, remove the white inner skin from the surface of the flesh, and wash it 2–3 times with tap water. Add ultrapure water at a ratio of 1:5, beat it with a blender, then wet grind it with a colloid mill, and sieve it through a 100-mesh screen. After sieving, the slurry naturally settles. Every 4 h, pour off the supernatant and the top layer of yellow substance, and wash it repeatedly with ultrapure water until the white sediment at the bottom is free of any discoloration. After purification, the water caltrop starch was dried in an oven at 35 °C and then milled to a 100-mesh size. The measured moisture contents of *T.qR‘Green’*, *T.qR‘Red’*, *T.a*, *T.bR‘Red’*, and *T.bR‘Green’* were, respectively, 11.36 ± 0.43%, 12.04 ± 0.51%, 12.15 ± 0.28%, 11.87 ± 0.42%, and 11.42 ± 0.26%; the protein contents were, respectively, 0.34 ± 0.02%, 0.44 ± 0.01%, 0.37 ± 0.00%, 0.31 ± 0.04%, and 0.40 ± 0.02%; and the fat contents were, respectively, 0.12 ± 0.01%, 0.14 ± 0.01%, 0.20 ± 0.03%, 0.17 ± 0.02%, and 0.12 ± 0.00%.

### 2.3. Amylose Content

The iodine colorimetric method was used to determine the amylose content (AC). The absorbance was measured at 635 nm using an ultraviolet spectrophotometer.

### 2.4. Structural Characteristics

#### 2.4.1. Normal Light Microscopy and Polarized Light Microscopy

A drop of starch solution with a mass fraction of 5% was placed on the slide and covered with a coverslip. The morphologies of the samples were observed using a light microscope (LV-VEPI-N, Nikon Instruments Co., Ltd., Shanghai, China) under both normal and polarized light at a magnification of 200×.

#### 2.4.2. Scanning Electron Microscopy (SEM)

A small amount of starch was taken and attached to a double-sided carbon tape, then plated with gold ion sputtering. SEM (SU8010; Hitachi Ltd., Tokyo, Japan) was used to observe the surface microstructure at a magnification of 500×.

#### 2.4.3. Particle Size

Prepare a 5% starch emulsion and disperse it uniformly using a viscolizer. The particle size of the water caltrop starch was measured with a laser particle size analyzer (Zetasizer Nano, Malvern Instrument Co., Ltd., Worcestershire, UK).

#### 2.4.4. X-Ray Diffraction (XRD)

Crystalline structure was conducted using an XRD instrument (Empyrean, PANalytical B.V., Almelo, The Netherlands). The device was operated at a scanning voltage of 40 kV and a current of 40 mA to measure the angle 2θ. The scanning range was set from 5° to 40°, with a scanning speed of 5°/min. The calculation steps for the relative crystallinity (RC) of starch were as follows: (1) The XRD spectra was baseline-corrected by drawing a tangent line between 2θ = 5–35° using PeakFit software version 4.12, and the area under this baseline was recorded as the amorphous region area (Aa); (2) The baseline-corrected spectra was subjected to peak fitting using a Gaussian function, and the sum of the areas of all fitted peaks was recorded as the crystalline region area (Ac); (3) The RC of the starch particles was calculated using the following formula [14].

(1) $$\mathrm{RC}\;(\%)=\frac{\mathrm{Ac}}{\mathrm{Aa}+\mathrm{Ac}}\;\times \;100$$

#### 2.4.5. Fourier Transform Infrared Spectroscopy (FT-IR)

The infrared spectral properties of the sample were measured using FTIR (IR-Tracer-100, Shimadzu Corp., Kyoto, Japan). Starch samples and KBr were weighed in a mass ratio of 1:100, ground, and pressed into pellets. The measurement was performed in transmission mode with a wavenumber range of 4000–400 cm^−1^, a resolution of 4 cm^−1^, and 64 scans. Baseline correction, smoothing, and deconvolution of the absorbance spectra were performed in the wavenumber range of 1200–800 cm^−1^. The peak intensity ratios (R_1047/1022_) between 1047 cm^−1^ and 1022 cm^−1^ and (R_995/1022_) between 995 cm^−1^ and 1022 cm^−1^ were calculated to evaluate the short-range molecular order and double helical structure of starch.

#### 2.4.6. Small-Angle X-Ray Scattering (SAXS)

The lamellar structure of the water caltrop starch sample was analyzed using a SAXS instrument (SAXSess mc2, Anton Paar, Graz, Austria). A monochromatic X-ray beam with a wavelength of λ Cu Kα = 1.54 Å was monitored by a photomultiplier tube. The copper rotating anode was operated at 50 kV and 30 mA. Scattering vector q (q = 4πsin2θ/λ, where λ was the wavelength and 2θ was the scattering angle) in the range 0.02–0.20 Å^−1^ was detected. Data were processed with Origin2021 software to obtain peak intensity (Imax), peak position (Smax), and peak area with the graphical method of Yuryev et al. [15]. Based on Woolf–Bragg’s equation, the semi-crystalline lamellar thickness (D, nm) was calculated.

(2) D=2π/Smax

For fractal structure analysis, SAXS curves were fitted with the power-law equation.

(3) $$I=Kq^{\alpha }$$

where K was a constant, and I, q, and α were scattering intensity, scattering vector, and power-law index, respectively. When −4 < α < −3, the sample can be judged as having a surface fractal structure, and the fractal dimension (Ds) can be calculated as Ds = α + 6. When −3 < α < −1, the sample can be judged as having a mass fractal structure, and the fractal dimension (Dm) can be calculated as Dm = −α [16].

#### 2.4.7. ^13^C CP/MAS Nuclear Magnetic Resonance (NMR) Spectroscopy

The ^13^C CP/MAS NMR spectra of water caltrop starch samples were acquired at room temperature on a 400 MHz WB Solid State NMR Spectrometer with a 4 mm CP/MAS detection probe (AVANCE III 400 WB, Bruker BioSpin GmbH, Rheinstetten, Germany). The test parameters were as follows: operating frequency of 100.62 MHz, acquisition time of 0.016 s, sample contact time of 1.8 ms, cumulative scans of 1600 times, delay time of 2 s, and spin frequency of 6 kHz.

#### 2.4.8. Amylopectin Chain Length Distributions

Standard Solution Preparation: Weigh 5 mg of each DP4-DP7 oligosaccharide from the standard kit and suspend in 5 mL of double-distilled water. Heat in a boiling water bath for 60 min with vortex mixing. Transfer 2.5 mL to a new centrifuge tube, add 125 μL sodium acetate, 5 μL NaN_3_, and 5 μL isoamylase. Incubate at 38 °C for 24 h. Then, transfer 600 μL to a new tube, evaporate under nitrogen, dissolve in 600 μL mobile phase, centrifuge at 8000 rpm for 5 min, and collect the supernatant. Sample Extraction: Weigh 5 mg of purified water caltrop starch and suspend it in 5 mL of double-distilled water. Heat the mixture in a boiling water bath for 60 min with intermittent vortexing. Take 2.5 mL of the gelatinized sample, add 125 μL of sodium acetate, 5 μL of NaN_3_, and 5 μL of isoamylase, then incubate at 38 °C for 24 h. Transfer 600 μL to a new centrifuge tube, evaporate under nitrogen at room temperature, dissolve the residue in 600 μL of the mobile phase, centrifuge at 12,000 rpm for 5 min, and collect the supernatant.

The analytical setup utilized in this study involved a Thermo ICS500 + ion chromatography system (ICS500+, Thermo Fisher Scientific, Waltham, MA, USA) featuring a Dionex™ CarboPac™ PA10 analytical column (250 mm × 4.0 mm, 10 μm). A 20 μL injection volume was employed with a mobile phase comprising 200 mM NaOH (Eluent A) and a blend of 200 mM NaOH/200 mM NaAC (Eluent B). The column temperature was maintained at 30 °C and monosaccharide constituents were identified using an electrochemical detector.

### 2.5. Physicochemical Properties

#### 2.5.1. Differential Scanning Calorimetry (DSC)

The thermal behavior of water caltrop starch samples was measured using a DSC instrument (DSC2000, TA Instruments, New Castle, DE, USA). Samples (3 mg) were equilibrated with ultrapure water (9 μL) in a sealed aluminum dish at 25 °C for 24 h. Scans were then performed from 30 °C to 100 °C (10 °C/min), and the onset temperature (To), peak temperature (Tp), conclusion temperature (Tc), and endothermic enthalpy (ΔH) were determined from each analysis using TA Universal Analysis 2000 software.

#### 2.5.2. Pasting Properties

The pasting properties of water caltrop starches were assessed using a Rapid Visco Analyzer (RVA-Super4, Perten Ruihua Scientific Instruments Co., Ltd., Beijing, China). A sample weighing 3 g (dry basis) was mixed with 25 mL of ultrapure water in an aluminum RVA tank. The mixture was heated to 50 °C for 1 min, further heated to 95 °C, and maintained at 95 °C for 2.5 min before being cooled back to 50 °C and held at this temperature for 2 min. Key parameters such as pasting temperature (PT), peak viscosity (PV), trough viscosity (TV), final viscosity (FV), breakdown (BD), and setback (SB) were determined during the analysis.

#### 2.5.3. Swelling Power and Solubility

The water caltrop starch suspension with a concentration of 2% (*w*/*v*) was subjected to heating and vibration in a water bath at temperatures of 65, 75, 85, and 95 °C for a duration of 30 min, followed by cooling to room temperature. Subsequently, the suspension underwent centrifugation at 3000 r/min for 15 min. The resulting supernatant was transferred to a Petri dish and dried at 105 °C until a constant weight was achieved. The solubility (S) and swelling power (SP) were determined using the following equations:

(4) $$S\;(\%)\;=\;\frac{A}{W}\;\times \;100\;$$

(5) $$\mathrm{SP}\;(g/g)=\frac{P}{W\;\times \;(S\;-\;100)}$$

where A, W, and P represent the weight of the dry supernatant, the weight of the initial dry sample, and the weight of the wet precipitate, respectively.

### 2.6. In Vitro Digestibility

Starch digestibility was assessed following the protocol by Englyst et al. [17] with adaptations. Starch samples (200 mg, dry weight) were dissolved in 15 mL of sodium acetate buffer (pH = 5.2) and mixed with 5 mL of a freshly prepared enzyme solution. Enzymatic hydrolysis was conducted in an oscillating water bath (150 rpm) at 37 °C for varying durations. To halt enzymatic activity, 0.5 mL of the hydrolyzed starch solution was mixed with 4 mL of anhydrous ethanol after 20 or 120 min of hydrolysis. Subsequently, centrifugation at 4000 rpm for 15 min was performed, and the resulting samples were analyzed for glucose content using a glucose oxidase/peroxidase (GOPOD) assay kit. The glucose levels were adjusted by a factor of 0.9 to determine the proportion of hydrolyzed starch. The subsequent equations were employed to quantify rapidly digestible starch (RDS), slowly digestible starch (SDS), and resistant starch (RS):

(6) $$\mathrm{RDS}\;\left(\%\right)\;=\;\frac{(G20\;-\;G0)\;\times \;0.9}{\mathrm{TG}}\;\times \;100$$

(7) $$\mathrm{SDS}\;(\%)=\frac{(G120\;-\;G20)\;\times \;0.9}{\mathrm{TG}}\;\times \;100\;$$

(8) RS (%)=100 − RDS (%) − SDS (%)

where TG denotes the total starch content, G0 is the free glucose content (mg) in the starch suspension, and G20 and G120 are the glucose contents (mg) of the starch hydrolysate after 20 and 120 min of hydrolysis, respectively.

### 2.7. Statistical Analysis

The experiments were carried out in triplicate, and the results are expressed as mean values ± standard deviations. Statistical analyses were conducted using SPSS 26 software. A one-way analysis of variance (ANOVA) and Duncan’s multiple range test were employed to evaluate significant differences among the data from different treatments. Furthermore, Pearson’s correlation was computed using Origin 2021. A *p*-value below 0.05 was deemed statistically significant.

## 3. Results

### 3.1. Amylose Content

The amylose content has a significant impact on the physicochemical properties of starch. As shown in Table 1, considerable variation exists among the water caltrop starch samples, with the green water caltrop, such as *T.qR‘Green’*, exhibiting the highest amylose content at 30.93%. The red water caltrop varieties, including *T.qR‘Red’* and *T.bR‘Red’*, demonstrate intermediate amylose content, and *T.a* shows the lowest amylose content at 25.37%. These differences may be attributed to the origin and variety of the water caltrops [18].

### 3.2. Structural Characteristics

#### 3.2.1. Granule Morphology

**Figure 1:**
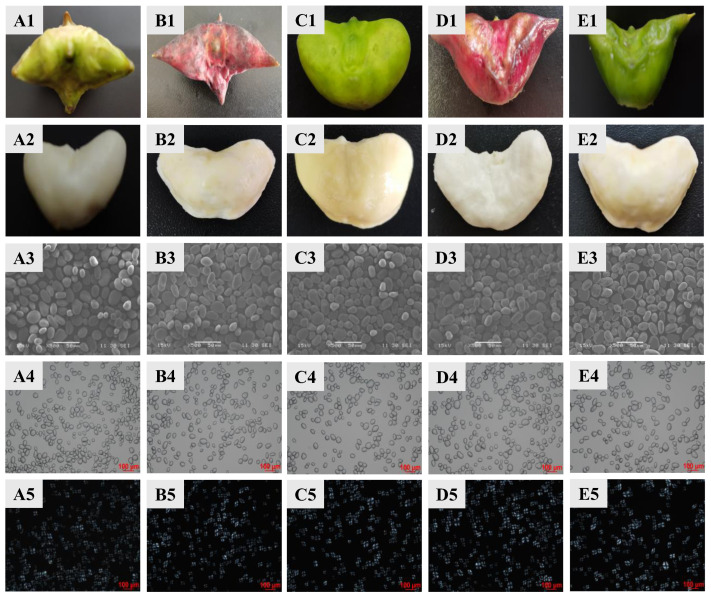
Photographs of water caltrop (1) and water caltrop flesh (2); morphologies of starch granules under scanning electron microscopy (3), normal light microscopy (4), and polarized light microscopy (5). (**A**) *T.qR‘Green’*; (**B**) *T.qR‘Red’*; (**C**) *T.a*; (**D**) *T.bR‘Red’*; (**E**) *T.bR‘Green’*.

With the genus *Trapa*, fruit morphology serves as a reliable classification criterion, particularly regarding fruit dimensions and spine number [19]. As shown in Figure 1, the five varieties of water caltrops exhibit distinct morphological characteristics. *T.qR‘Green’* and *T.qR‘Red’* both possess four horn-like protrusions, comprising two long conical spines and two short blunt spines, forming dimorphic spine pairs. *T.a* displays a smooth pericarp devoid of spines, with rounded protrusions at the fruit shoulders and an approximately elliptical shape. *T.bR‘Red’* and *T.bR‘Green’* possess a pair of elongated, acute spines, with their longitudinal axis significantly exceeding the transverse diameter.

The microscopic structures of starch granules from different varieties of water caltrop are shown in Figure 1. The starch granules vary in size, with larger granules exhibiting elliptical or polygonal shapes, while smaller granules appear spherical. All surfaces are smooth and unwrinkled, consistent with tuber starch’s typical morphology. Notably, *T.bR‘Green’* starch granules are significantly larger than those of other varieties. This confirms that granule size and shape are variety-specific traits. Under polarized light, all five varieties of water caltrop starch exhibit clear birefringence, displaying distinct Maltese crosses. The hilum is consistently centralized across granules, with no significant observable interspecific variations in Maltese crosses position or morphology.

#### 3.2.2. Particle Size

**Figure 2:**
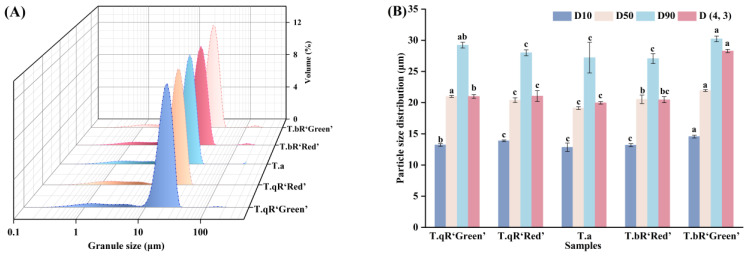
The particle size distribution (**A**) and particle size parameters (**B**) of all water caltrop starch samples.

The terms D10, D50, and D90 denote the particle diameters representing the accumulations of 10%, 50%, and 90% of granules, respectively. Furthermore, D(4, 3) signifies the volume-based mean particle diameter of starch granules [20]. The particle size distribution and parameters of starch samples from different water caltrop varieties are shown in Figure 2. The particle size distribution curves exhibited unimodal distributions with a primary peak between 16 and 35 μm, indicating concentrated granule distribution with the typical size range for tuber starches. Notably, a minor peak beyond 100 μm was observed in all five varieties, representing very large particles with minimal proportions, likely attributed to secondary aggregation of starch granules [21]. Among the five varieties, *T.bR‘Green’* starch displayed the largest granules, D(4, 3) value reached 28.28 μm, which is higher than that of other varieties (18.34–22.15 μm), consistent with the granule sizes observed via SEM.

#### 3.2.3. Crystalline Structure

**Figure 3:**
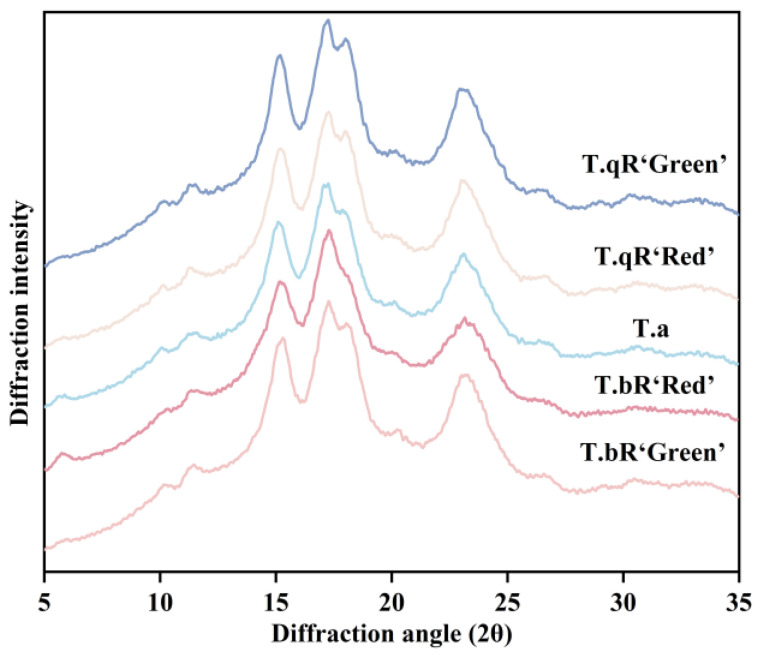
XRD patterns of all water caltrop starch samples.

The XRD analysis (Figure 3) determined the crystal structures of starch samples obtained from various water caltrop varieties, revealing notable distinctions in crystalline forms among the samples. *T.qR‘Green’*, *T.qR‘Red’*, and *T.bR‘Green’* displayed diffraction peaks at 15° and 23°, with additional double peaks around 17° and 18°, which are characteristic of A-type crystalline starch [20]. In comparison, *T.bR‘Red’* and *T.a* exhibited C-type crystalline (a superposition of A- and B-type crystalline structure). Typically, the A-type polymorph and B-type polymorph are constructed by monoclinic and hexagonal crystalline unit cells, respectively [22]. Based on the proportion of A-type polymorph and B-type polymorph, three types of C-type patterns were classified, i.e., C/A-type (showing diffraction peak at 5.6°, 15°, 17°, 18°, and 23° 2θ), C/B-type (showing diffraction peak at 5.6°, 15°, 17°, 22°, and 24° 2θ), and C-type (typical C-type, showing diffraction peak at 5.6°, 15°, 17°, and 23° 2θ) [23]. *T.bR‘Red’* can be further classified as a typical C-type, while *T.a* is of the C/A-type.

There are differences in crystallinity among starches from different water caltrop varieties (Table 1), with relative crystallinity ranging from 24.94% to 29.04%. Among them, *T.bR‘Green’* exhibited the highest crystallinity (29.04%), followed by *T.qR‘Green’* (27.60%), *T.a* (27.14%), and *T.qR‘Red’* (26.76%), while *T.bR‘Red’* showed the lowest crystallinity (24.94%). These variations are strongly correlated with the starch granule morphology. Scanning electron microscopy analysis demonstrated that *T.bR‘Green’* exhibited the largest granule size, aligning with the correlation between particle size and crystallinity as suggested by Zhang et al. [24]. Subsequent investigations have revealed that inherent molecular characteristics contribute to these distinctions; elevated amylose levels impede the structured organization of amylopectin double helices, while an increased ratio of short chains within amylopectin facilitates the development of compact crystalline domains. Additionally, environmental factors in aquatic habitats (e.g., water temperature, light intensity) may influence amyloplast development, ultimately altering the pathway for crystalline structures formation.

#### 3.2.4. Short-Range Ordered Structure

**Figure 4:**
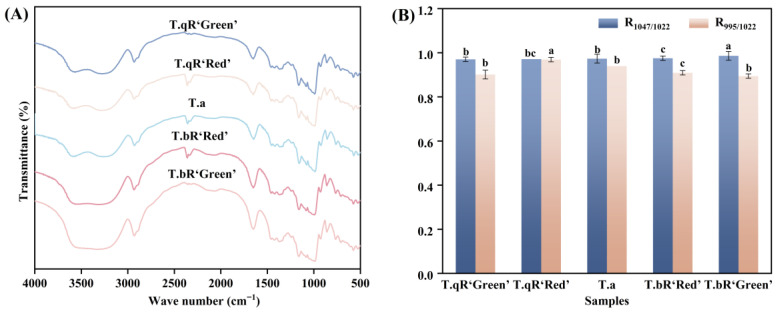
FTIR patterns (**A**), the R_1047/1022_ and R_995/1022_ values (**B**) of all water caltrop starch samples.

The FT-IR spectra of starch samples from different varieties of water caltrop are presented in Figure 4. The FT-IR spectra of starches from different water caltrop varieties are similar. The spectral bands within the 1200–800 cm^−1^ range represent stretching and bending vibrations of various chemical bonds, predominantly involving C-C and C-O stretching vibrations, along with C-H-O bending vibrations. Variations in peak morphology can effectively indicate distinctions in the short-range structural organization of starch molecules. Specifically, the peaks observed at 995 cm^−1^ and 1047 cm^−1^ are linked to the helical structural properties and molecular arrangement of starch, while the vibrational mode at 1022 cm^−1^ corresponds to the disordered or amorphous phase [25,26]. Consequently, the R_1047/1022_ and R_995/1022_ ratios are commonly employed to assess the level of organization and double-helical configuration of starch [27].

The R_1047/1022_ and R_995/1022_ values exhibited significant variations among starch samples from different water caltrop starch varieties (Figure 4), reflecting inherent structural differences in their starches. The R_1047/1022_ values of water caltrop starches ranged from 0.970 to 0.986, with *T.bR‘Green’* (0.986) exhibiting the highest short-range order, while *T.qR‘Green’* (0.970) showed the lowest. This result aligns with the XRD analysis. *T.bR‘Green’* also displayed the highest crystallinity (29.04%). These findings indicate a positive correlation between long-range crystalline order and short-range molecular arrangement. In contrast, the R_995/1022_ ratio followed a different trend, with *T.qR‘Red’* (0.969) and *T.bR‘Green’* (0.894) having the highest and lowest values, respectively. This suggests that the distribution of side-chain lengths in amylopectin may influence the helical content in starch by affecting the packing density of double helices.

#### 3.2.5. Lamellar Structure

**Figure 5:**
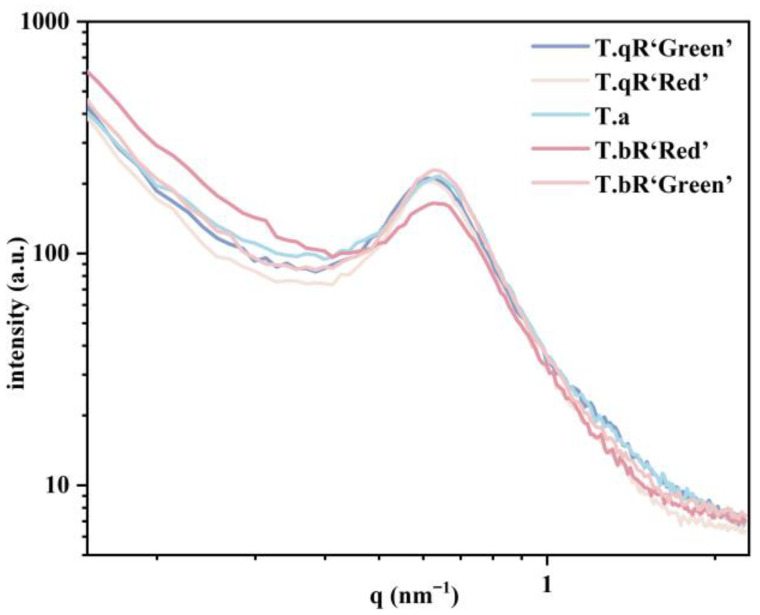
Double-logarithmic SAXS patterns of all water caltrop starch samples.

The double-logarithmic SAXS patterns of starch samples from different water caltrop varieties are shown in Figure 5. All starch SAXS patterns exhibited a characteristic scattering peak at a q-value of approximately 0.6 nm^−1^. The D values for the starches are listed in Table 2. The D values of water caltrop starches ranged from 9.81 to 10.27 nm, consistent with the semi-crystalline lamellar thickness previously reported for starches from other plant sources such as blue wheat starch, maize starch, and lotus seed starch [28,29,30].

The peak area is positively correlated with the degree of order in the lamellar regions [31]. The order of peak area magnitude was *T.bR‘Green’* > *T.qR‘Green’* > *T.qR‘Red’* > *T.a* > *T.bR‘Red’* (Table 2), indicating that *T.bR‘Green’* and *T.bR‘Red’* possessed the highest and lowest lamellar order, respectively, consistent with the XRD results. Moreover, the peak intensity is contingent upon the level of ordered semi-crystalline structure and/or the electron density contrast (Δρ) between the crystalline and amorphous lamellae compared to the amorphous background [12]. Significantly divergent peak intensities were observed across various water caltrop starch varieties, suggesting notable distinctions in their semi-crystalline architectures.

The results showed that the power-law exponent (α) of water caltrop starches ranged between −3 and −1, signifying a mass fractal (Dm) structure, which suggests the samples possess self-similarity in their density distribution. The mass fractal dimension (Dm) values ranged from 2.50 to 2.88 (corresponding to α = −2.50 to −2.88). A smaller Dm value indicates a denser aggregate structure within the granule [16]. Among them, *T.a* and *T.bR‘Red’* exhibit a lower Dm value, indicating a dense three-dimensional network structure within their particles. In contrast, *T.qR‘Green’*, *T.qR‘Red’*, and *T.bR‘Green’* show a higher Dm value, suggesting a looser fractal aggregation state.

#### 3.2.6. Helical Structure

**Figure 6:**
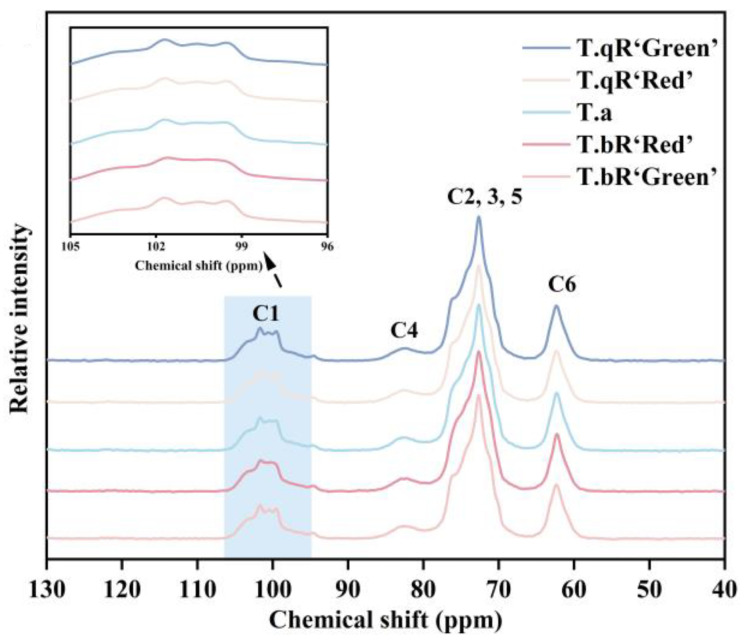
^13^C CP/MAS NMR patterns of all water caltrop starch samples.

Figure 6 displays the ^13^C CP/MAS NMR spectra of starch samples obtained from various water caltrop varieties. The resonance at 61.8 ppm is assigned to C6, while the broad signal ranging from 68 to 78 ppm collectively corresponds to the C2, C3, and C5 sites. Peaks observed at 81.8 ppm are indicative of the C4 site, and resonances falling within the 100–103 ppm range are associated with the C1 site [32,33]. Differences in peak shapes within the C1 region among the varieties can effectively distinguish crystal types of the starch. The multiplicity of the C1 resonance corresponds to the packing type of the starch granule. The C1 peak of spectrum is a triplet for A-type starch, and a doublet (approximately 101 and 100 ppm) for B-type starch. In general, the C-type starch also shows triplet C1 spectrum if the A-type crystalline structure is predominant in the sample, and doublet C1 spectrum if the B-type crystalline structure is predominant. The *T.qR‘Green’*, *T.qR‘Red’,* and *T.bR‘Green’* samples occurred as typical triplets at about 99.6, 100.5, and 101.7 ppm, indicating that all three were typical A-type starches. *T.a* sample showed inconspicuous triplets and *T.bR‘Red’* presented inconspicuous doublet. These results in combination with XRD patterns further showed that starches from *T.a* was C/A-type crystal and *T.bR‘Red’* was C-type crystal [34].

#### 3.2.7. Fine Structure of Amylopectin

**Figure 7:**
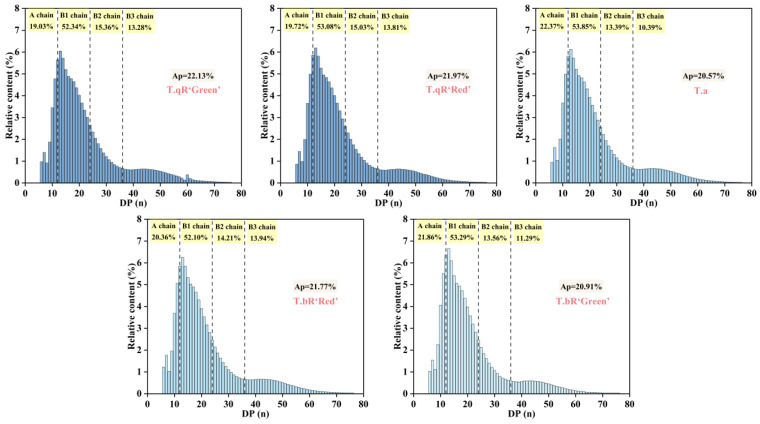
Amylopectin chain length distributions of all water caltrop starch samples. Ap is the average chain length of the amylopectin chain.

Amylopectin’s chain length distribution is divided into four fractions based on the degree of polymerization (DP) as follows: A-chains (DP = 6–12), B1-chains (DP = 13–24), B2-chains (DP = 25–36), and B3-chains (DP > 36) [35]. A-chains, which are the outermost chains connected to B-chains through α-1,6 glycosidic bonds, are incapable of further branching. Short chains include A- and B1-chains, while long chains encompass B2- and B3-chains, which have a propensity to form double-helical structures [36]. Figure 7 illustrates the amylopectin chain length distributions in starch samples derived from various water caltrop cultivars. The data reveal a consistent pattern across all samples, characterized by two prominent peaks at degrees of DP 13 and 44. The B1 (DP = 13–24) accounted for the highest proportion of amylopectin chain length distributions in all water caltrop starches, which is consistent with findings for pea starch [37] and buckwheat starch [38]. The proportion of short chains (A + B1) in *T.a* reached 76.22%, which was significantly higher than that in *T.qR‘Green’* (71.37%). Notably, the total long chain (B2 + B3) content in *T.qR* varieties (28.64–28.84%) exceeded that in *T.bR* varieties (24.85–28.15%). This suggests a potential correlation between the morphological characteristics of water caltrops and the regulatory mechanisms underlying starch biosynthesis. Amylopectin with a chain length of DP 13–24 was more likely to form the crystalline structure of starch. *T.a* had the lowest relative crystallinity, which may be due to the lower proportion of DP 13–24. However, the content of amylopectin B1 chain was not the only factor affecting the crystallinity. Differences in the size and arrangement of crystals in amylopectin cells and the interactions between crystals may also lead to differences in crystallinity [39].

### 3.3. Physicochemical Properties

#### 3.3.1. Thermal Properties

**Figure 8:**
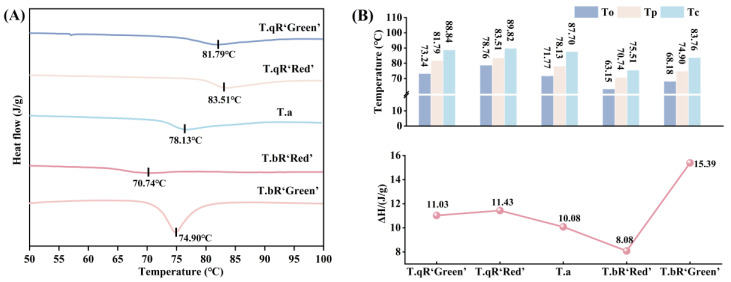
DSC patterns (**A**) and thermal properties parameters (**B**) of all water caltrop starch samples.

The thermal properties of starches from different water caltrop varieties are shown in Figure 8. The To, Tp, and Tc ranged from 63.15 to 78.76 °C, 70.74–83.51 °C, and 75.51–89.82 °C, respectively, while ΔH varied between 8.10 and 15.39 J/g, respectively. The order of To, Tp, and Tc among the five water caltrop starches was *T.qR‘Red’* > *T.qR‘Green’* > *T.a* > *T.bR‘Green’* > *T.bR‘Red’*. Studies have demonstrated that longer amylopectin chains tend to form more stable double-helical structures, leading to higher gelatinization temperatures [40,41]. Consistent with the long-chain amylopectin proportions reported in the fine structure of amylopectin, *T.qR‘Red’* had the highest long chains content, which explains why it exhibited the highest gelatinization temperatures (To, Tp, and Tc), directly supporting this mechanistic link between chain structure and thermal properties.

The ΔH reflects the energy needed to disrupt the intermolecular bonds within starch, which is associated with its organized physical arrangement, including its crystalline structure [42,43]. Significant differences in ΔH were observed among the different water caltrop starch varieties. *T.bR‘Green’* exhibited the highest ΔH, followed by *T.qR‘Red’* and *T.qR‘Green’*, then *T.a*, with *T.bR‘Red’* showing the lowest value. Notably, the ΔH values aligned with the crystallinity of starch granules. *T.bR‘Green’* and *T.bR‘Red’,* respectively, have the highest and lowest crystallinity, and their ΔH values are correspondingly the highest and lowest, confirming that the stability of crystalline structures directly influences the energy required for gelatinization [44].

#### 3.3.2. Pasting Properties

**Figure 9:**
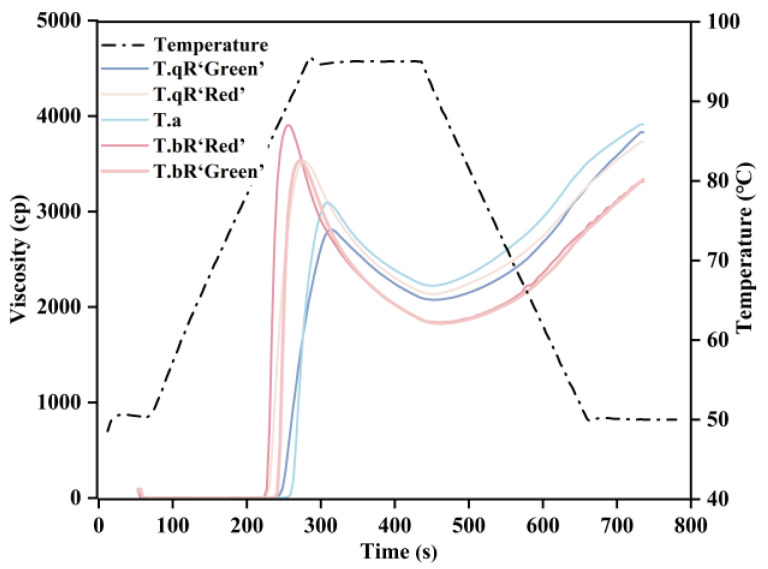
RVA patterns of all water caltrop starch samples.

The gelatinization curves of starch samples from different varieties of water caltrop are shown in Figure 9, with the corresponding gelatinization parameters presented in Table 3. The PV of the five varieties ranged between 2850.33 and 3963.33 cp, with *T.bR‘Red’* exhibiting the highest PV and *T.qR‘Green’* the lowest. Notably, *T.qR‘Green’* had the lowest short-chain content in amylopectin and the highest amylose content, structural features that directly influence PV. Amylopectin short chains are more prone to leaching from starch granules during heating or processing, which significantly increases the PV of starch [43]. In contrast, the linear structure of amylose effectively inhibits starch granules’ swelling during heating, reducing granule rupture from excessive swelling and ultimately maintaining PV at a lower level [40,41]. The BD value reflects the tendency of swollen starch granules to rupture under continuous high-temperature shearing, serving as an indicator of thermal stability. A lower BD signifies stronger heat resistance [45,46]. Among the five water caltrop starch varieties, BD ranged from 747.00 to 2062.67 cp, with *T.bR‘Red’* showing the highest BD and *T.qR‘Green’* the lowest. This result directly confirms that *T.qR‘Green’* starch granule possess superior thermal stability. The SB value reflects the tendency of starch retrogradation. SB values for the five water caltrop starch varieties ranged from 1479.33 to 1757.33 cp, with *T.bR‘Red’* exhibiting the lowest SB. This suggests that *T.bR‘Red’* has superior anti-retrogradation properties. The SB of *T.qR‘Green’* was the highest, which was related to its high amylose content. This is because amylose is more prone to rapid recrystallization, thereby accelerating the retrogradation of the system [42].

#### 3.3.3. Swelling Power and Solubility

**Figure 10:**
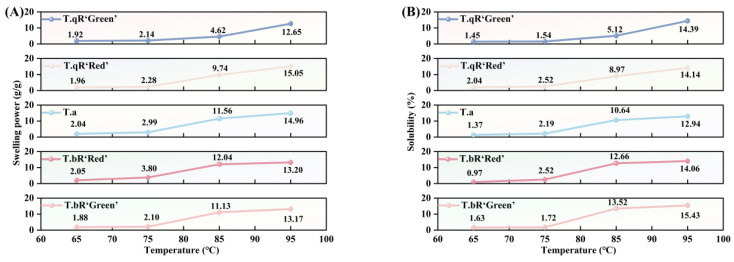
Swelling power (**A**) and solubility (**B**) of all water caltrop starch samples.

SP and S are key indicators of the water-holding capacity and internal binding ability of starch granules [47]. As shown in Figure 10, both swelling power and solubility of all water caltrop starch samples increased with temperature from 65 °C to 95 °C, albeit at a gradually decreasing rate. When heated in excess water, hydrogen bonds between starch molecules are progressively disrupted, leading to partial breakdown of the crystalline structure within the granules. This structural loosening allows water molecules to penetrate the granule matrix and form hydrogen bonds with exposed hydroxyl groups in amylose and amylopectin, ultimately enhancing swelling power and solubility [48]. The temperature rise led to a gradual reduction in the rate of increase in swelling power and solubility. This slowdown is attributed to the intermolecular entanglement and cross-linking of amylopectin chains, resulting in the formation of a stable network structure. This structure restricts the further expansion of the granules [49].

Among the five types of water caltrop starch, *T.qR‘Green’* exhibited the lowest swelling power across 65–95 °C, which was consistent with its highest amylose content. Amylose can entangle with the internal segments of amylopectin in the amorphous lamellae of the granules, thereby inhibiting amylose leaching and restricting granule expansion [50]. Notably, a contrasting trend was observed for solubility; at 85 °C and 95 °C, *T.bR‘Green’* demonstrated the highest solubility among all varieties. This phenomenon can be attributed to the higher amylose content, which enhances the presence of amorphous regions within starch granules. Consequently, this facilitates the penetration of water molecules and structural disruption [13].

### 3.4. In Vitro Digestibility

**Figure 11:**
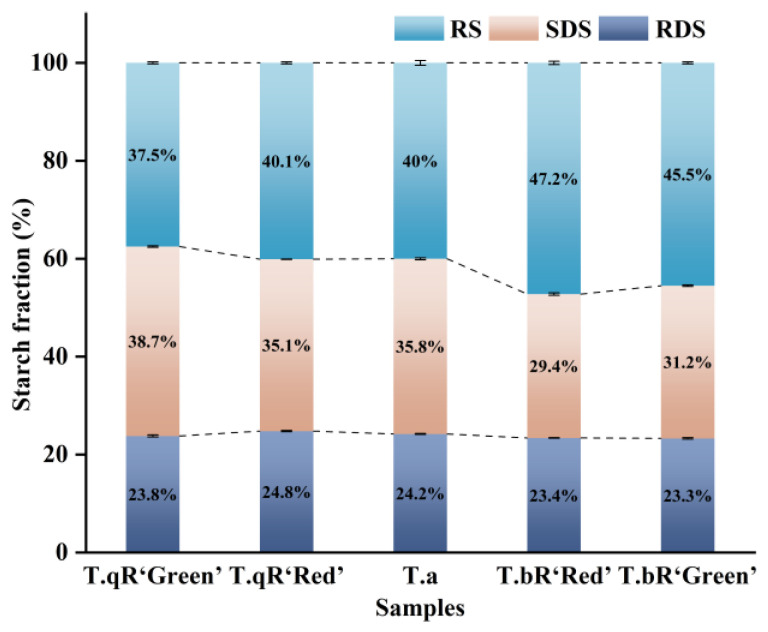
RDS, SDS, and RS contents of all water caltrop starch samples.

Figure 11 displays the RDS, SDS, and RS contents of five varieties of water caltrop starch, ranging from 23.3 to 24.8%, 29.3–39.4%, and 37.5–47.2%, respectively. While RDS content varied minimally across varieties, SDS and RS contents exhibited significant fluctuations. Notably, *T.bR‘Green’* displayed the highest amylose content and higher RS content. Amylose enhances intermolecular interactions with amylopectin, promoting the formation of a smoother, more rigid granule surface. This structural characteristic impedes enzymes’ diffusion and absorption onto starch granules, thereby reducing starch digestibility [18]. Additionally, *T.bR‘Green’* starch granules were the largest, resulting in a relatively low specific surface area. This reduced surface area limits contact with digestive enzymes and lowers digestibility (i.e., higher RS content). Moreover, *T.bR‘Green’* exhibited the highest crystallinity and short-range order, which restricts enzymatic hydrolysis and slows the digestion process, collectively leading to its lower digestibility and high RS content.

### 3.5. Correlation Analysis

**Figure 12:**
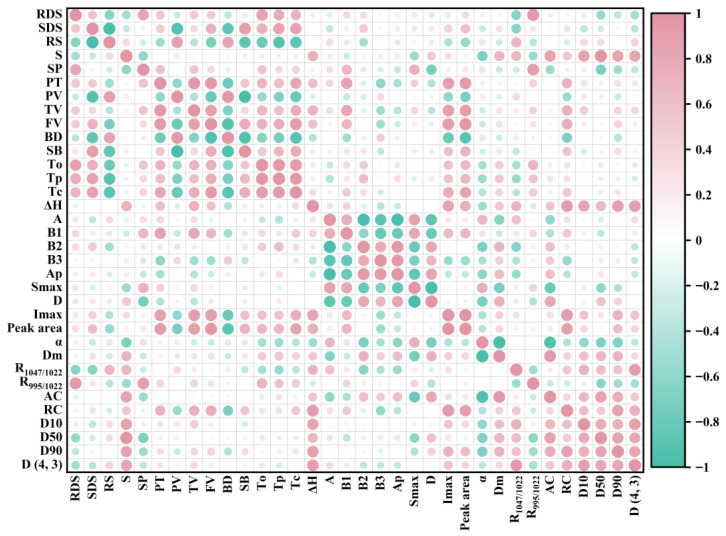
Correlation analysis between water caltrop starch structure and properties.

Through Pearson correlation analysis, the structure of water caltrop starches was systematically correlated with its properties to establish structure–property relationships, with results presented in Figure 12. Amylopectin chain length distribution exhibited significant associations with thermal properties. A positive correlation was observed between B1-chains and Tc (R = 0.50, *p* < 0.05). This correlation is consistent with the findings of Yang et al. [51], who demonstrated that an increased presence of B1-chains in amylopectin facilitates the development of a denser crystalline structure, which could lead to improving thermal stability. Meanwhile, the positive correlation between B2-chains and Tp (R = 0.60, *p* < 0.05) further suggests an impact of chain length on the thermal stability of starch crystallinity [43]. Moreover, the positive correlation between short-range molecular order (R_1047/1022_) and ΔH (R = 0.70, *p* < 0.05) implies that a greater degree of short-range order may necessitate increased energy for disruption.

Several significant correlations linked RC to pasting behavior. RC negatively correlated with PV (R = −0.55, *p* < 0.05). This could be because a compact crystalline structure restricts starch granules’ water absorption and swelling, thereby reducing PV. The negative correlation between RC and BD (R = −0.70, *p* < 0.05) suggests that higher crystallinity may confer enhanced shear resistance, resulting in less viscosity breakdown. Conversely, the positive correlation between RC and SB (R = 0.55, *p* < 0.05) is possibly related to the retrogradation process, which promotes molecular rearrangement. Amylopectin chain length also showed associations with pasting properties. B1-chains correlated positively with TV (R = 0.82, *p* < 0.05) and FV (R = 0.69, *p* < 0.05), while B3-chains showed negative correlations with TV (R = −0.53, *p* < 0.05) and FV (R = −0.53, *p* < 0.05). These results indicate that starch with higher B1 and lower B3 chain content tends to exhibit elevated TV and FV.

Additionally, the positive correlation between B1-chains and SP (R = 0.69, *p* < 0.05) is in agreement with the concept that short-chain amylopectin can enhance swelling power by facilitating hydrogen bond with water [52]. S exhibited significant positive correlations with D10 (R = 0.86, *p* < 0.05), D50 (R = 0.99, *p* < 0.05), D90 (R = 0.85, *p* < 0.05), and D(4, 3) (R = 0.85, *p* < 0.05), indicating that larger granules are associated with higher solubility. Additionally, AC was positively correlated with S (R = 0.86, *p* < 0.05) and negatively correlated with SP (R = −0.55, *p* < 0.05).

R_1047/1022_ was positively correlated with RS content (R = 0.69, *p* < 0.05) but negatively correlated with RDS content (R = −0.65, *p* < 0.05). This is consistent with the understanding that a higher short-range order restricts enzymatic hydrolysis, thereby slowing digestion [53]. Furthermore, the negative correlations of RDS with D50 (R = −0.57, *p* < 0.05) and D(4, 3) (R = −0.51, *p* < 0.05) support the view that larger granules, with a lower specific surface area, may reduce enzyme contact.

Inter-correlations among structural parameters were also observed. RC was positively correlated with B1-chains (R = 0.52, *p* < 0.05) and negatively correlated with B3-chains (R = −0.59, *p* < 0.05). These findings confirmed that short chains of amylopectin may contribute to structural defects in starch granules, while long chains enhance their stability. R_1047/1022_ showed positive correlations with particle size parameters (D10: R = 0.73; D50: R = 0.63; D90: R = 0.57; D(4, 3): R = 0.92; *p* < 0.05), suggesting that larger starch granules in water caltrop may possess a more ordered short-range structure. Finally, the negative correlation between Dm and A-chains (R = −0.66, *p* < 0.05) and its positive correlation with B2-chains (R = 0.71, *p* < 0.05) implies a potential relationship where shorter amylopectin chains are associated with greater granule compactness.

## 4. Discussion

The starch characteristics of five different varieties of water caltrop were examined systematically in this research. The results demonstrated that the starch granules predominantly exhibited elliptical and polygonal morphologies, with particle size following a unimodal distribution and an average diameter ranging from 19 to 29 μm. The amylose content varied between 25.37% and 30.93%. Regarding crystalline structure, *T.bR‘Green’*, *T.qR‘Green’*, and *T.qR‘Red’* displayed A-type crystal, while *T.bR‘Red’* exhibited C-type crystal, and *T.a* showed a C/A-type pattern. The crystallinity degree fluctuated between 24% and 30%, with *T.bR‘Green’* having the highest crystallinity. In terms of solubility and swelling properties, *T.qR* varieties exhibited lower solubility and swelling power, whereas *T.bR‘Green’* demonstrated the highest solubility among all varieties. Molecular structure analysis revealed that while the molecular structures of the different varieties were similar, *T.bR‘Red’* possessed a unique lamellar structure with a more ordered internal arrangement. The chain length distribution of amylopectin followed a consistent trend across all varieties, primarily concentrated at DP14-17. Among them, *T.qR’Green’* had the longest Ap and the lowest short chains (A + B1) content, while *T.a* contained the highest short chains content. Compared with other varieties, *T.qR* varieties showed higher long chains (B2 + B3) content. Thermal property studies indicated significant differences among the varieties. The *T.qR* varieties showed higher gelatinization temperatures, while the *T.bR* varieties exhibited lower values. *T.bR‘Green’* had the highest ΔH. Digestibility studies revealed that the *T.qR* varieties contained higher levels of SDS, with *T.qR‘Green’* being particularly notable. In contrast, the *T.bR* varieties had the highest RS content, indicating stronger resistance to digestion. These results provide valuable insights into the properties of water caltrop starch, offering a scientific basis for variety selection in developing water caltrop starch products for specific applications (such as food requiring high gel strength or health foods requiring slow digestion characteristics), thereby enhancing its potential for further utilization.

## Figures and Tables

**Table 1:** The amylose content and relative crystallinity of all water caltrop starch samples.

Samples	Amylose Content (%)	Relative Crystallinity (%)
*T.qR‘Green’*	30.93 ± 1.73 ^a^	27.60 ± 0.44 ^b^
*T.qR‘Red’*	30.10 ± 0.48 ^ab^	26.76 ± 0.61 ^b^
*T.a*	25.37 ± 0.42 ^b^	27.14 ± 0.16 ^b^
*T.bR‘Red’*	28.22 ± 0.63 ^ab^	24.94 ± 0.61 ^c^
*T.bR‘Green’*	30.71 ± 0.21 ^ab^	29.04 ± 0.42 ^a^

Data represent means ± standard deviations. For each column, values not displaying the same letter are significantly different (*p* < 0.05).

**Table 2:** Lamellar structural characteristics of all water caltrop starch samples.

Samples	Smax (nm^−1^)	D (nm)	Peak Area	Imax (a.u.)	α	Dm
*T.qR‘Green’*	0.612 ± 0.001 ^d^	10.27 ± 0.02 ^a^	34.66 ± 0.55 ^b^	145.03 ± 0.79 ^c^	−2.88 ± 0.04 ^a^	2.88 ± 0.04 ^a^
*T.qR‘Red’*	0.626 ± 0.001 ^bc^	10.04 ± 0.01 ^bc^	33.86 ± 0.57 ^bc^	147.64 ± 0.87 ^b^	−2.87 ± 0.09 ^a^	2.87 ± 0.09 ^a^
*T.a*	0.641 ± 0.000 ^a^	9.81 ± 0.00 ^d^	33.69 ± 0.45 ^c^	144.88 ± 0.54 ^c^	−2.50 ± 0.05 ^b^	2.50 ± 0.05 ^b^
*T.bR‘Red’*	0.624 ± 0.002 ^c^	10.07 ± 0.03 ^b^	21.25 ± 0.24 ^d^	93.43 ± 0.33 ^d^	−2.61 ± 0.04 ^b^	2.61 ± 0.04 ^b^
*T.bR‘Green’*	0.627 ± 0.001 ^b^	10.02 ± 0.02 ^c^	38.16 ± 0.43 ^a^	165.37 ± 0.42 ^a^	−2.81 ± 0.11 ^a^	2.81 ± 0.11 ^a^

Data represent means ± standard deviations. For each column, values not displaying the same letter are significantly different (*p* < 0.05). Smax, peak position; D, the semi-crystalline lamellar thickness; Imax, peak intensity; α, power-law index; Dm, mass fractal dimension.

**Table 3:** Pasting properties parameters of all water caltrop starch samples.

Samples	PT (°C)	PV (cP)	TV (cP)	FV (cP)	BD (cP)	SB (cP)
*T.qR‘Green’*	83.70 ± 0.36 ^b^	2850.33 ± 69.78 ^d^	2088.66 ± 12.05 ^c^	3873.33 ± 37.85 ^b^	747.00 ± 9.84 ^d^	1757.33 ± 6.65 ^a^
*T.qR‘Red’*	85.88 ± 0.59 ^a^	3456.67 ± 35.11 ^b^	2286.67 ± 14.64 ^a^	3868.00 ± 7.54 ^b^	1193.00 ± 6.08 ^b^	1589.33 ± 9.01 ^c^
*T.a*	86.55 ± 0.60 ^a^	3081.00 ± 26.88 ^c^	2233.33 ± 10.69 ^b^	3927.00 ± 30.44 ^a^	874.00 ± 5.29 ^c^	1681.33 ± 6.65 ^b^
*T.bR‘Red’*	79.40 ± 0.52 ^c^	3963.33 ± 56.86 ^a^	1867.00 ± 30.01 ^d^	3316.67 ± 6.42 ^c^	2062.67 ± 6.65 ^a^	1479.33 ± 9.07 ^d^
*T.bR‘Green’*	85.63 ± 0.40 ^a^	3476.67 ± 20.81 ^b^	2270.33 ± 19.00 ^a^	3844.67 ± 19.55 ^b^	1182.00 ± 6.08 ^b^	1596.00 ± 5.29 ^c^

Data represent means ± standard deviations. For each column, values not displaying the same letter are significantly different (*p* < 0.05).

## Data Availability

The original contributions presented in this study are included in the article. Further inquiries can be directed to the corresponding authors.
